# Associations of long-term exposure to PM_1_, PM_2.5_, NO_2_ with type 2 diabetes mellitus prevalence and fasting blood glucose levels in Chinese rural populations

**DOI:** 10.1016/j.envint.2019.105213

**Published:** 2019-12

**Authors:** Feifei Liu, Yuming Guo, Yisi Liu, Gongbo Chen, Yuxin Wang, Xiaowei Xue, Suyang Liu, Wenqian Huo, Zhenxing Mao, Yitan Hou, Yuanan Lu, Chongjian Wang, Hao Xiang, Shanshan Li

**Affiliations:** a Department of Global Health, School of Health Sciences, Wuhan University, 115# Donghu Road, Wuhan, China; b Department of Epidemiology and Preventive Medicine, School of Public Health and Preventive Medicine, Monash University, Melbourne, Australia; c Department of Epidemiology and Biostatistics, School of Public Health, Zhengzhou University, Zhengzhou, Henan, China; d Department of Environmental and Occupational Health Sciences, University of Washington, 1959 NE Pacific Street, Seattle, USA; e Environmental Health Laboratory, Department of Public Health Sciences, University Hawaii at Manoa, 1960 East West Rd, Biomed Bldg, D105 Honolulu, USA

**Keywords:** Air pollution, Type 2, Prevalence, Fasting blood glucose, Rural health

## Abstract

**Objectives:**

To evaluate the associations between long-term exposure to particulate matter with an aerodynamic diameter ≤1.0 μm and ≤2.5 μm (PM_1_ and PM_2.5_), nitrogen dioxide (NO_2_) and type 2 diabetes prevalence and fasting blood glucose levels in Chinese rural populations.

**Material and methods:**

A total of 39, 259 participants were enrolled in The Henan Rural Cohort study. Questionnaires and medical examination were conducted from July 2015 to September 2017 in rural areas of Henan province, China. Three-year average residential exposure levels of PM_1_, PM_2.5_, NO_2_ for each subject were estimated by a spatiotemporal model. Logistic regression and linear regression models were applied to estimate the associations between PM_1_, PM_2.5_, NO_2_ exposure and type 2 diabetes prevalence and fasting blood glucose levels.

**Results:**

The mean 3-year residential exposure concentrations of PM_1_, PM_2.5_ and NO_2_ was 57.4 μg/m^3^, 73.4 μg/m^3^ and 39.9 μg/m^3^, respectively. Higher exposure concentrations of PM_1_, PM_2.5_, NO_2_ by 1 μg/m^3^ was positively related to a 4.0% (95%CIs: 1.026, 1.054), 6.8% (1.052, 1.084) and 5.0% (1.039, 1.061) increase in odds of type 2 diabetes in the final adjusted models. Besides, a 1 μg/m^3^ increase of PM_1_, PM_2.5_ and NO_2_ was related to a 0.020 mmol/L (95%CIs: 0.014, 0.026), 0.036 mmol/L (95%CIs: 0.030, 0.042) and 0.030 mmol/L (95%CIs: 0.026, 0.034) mmol/L higher fasting blood glucose levels.

**Conclusions:**

Higher exposure concentrations of air pollutants were positively related to the increased odds of type 2 diabetes, as well as higher fasting blood glucose levels in Chinese rural populations.

## Highlights

• Higher PM_1_, PM_2.5_, NO_2_ exposure concentrations were associated with increased odds of type 2 diabetes.
• Higher levels of PM_1_, PM_2.5_, NO_2_ exposure were associated with an elevated fasting blood glucose levels.
• Males and populations aged 65 years or older may susceptible to air pollution.

## Graphical abstract

**Figure:**
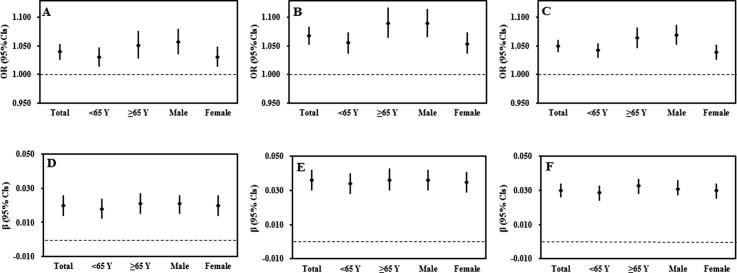

## 1 Introduction

Diabetes is the main cause of the increasing premature deaths and global disease burden (GBD, 2016). It was reported that in 2015, approximately 415 million people were diagnosed with diabetes, and nearly 5.0 million of deaths were due to diabetes worldwide. Besides, the estimated global total healthcare expenditure on diabetes was 673 billion US dollars (Ogurtsova et al., 2017). It is also estimated that there will be 642 million diabetics worldwide by 2040 (Ogurtsova et al., 2017). In addition, almost 95% of diabetic patients are diagnosed as type 2 diabetes (Hernandez et al., 2018, Morrison et al., 2019). Known risk factors for developing type 2 diabetes include genetics, aging, high body mass index (BMI) and unhealthy diet. Evidence from published studies illustrates that environmental factors may also play important roles in type 2 diabetes development (Liu et al., 2016, Liu et al., 2019a).

Ambient air pollution is a serious public health issue worldwide, and significantly contributes to the global disease burden (Ogurtsova et al., 2017). Recently, studies identifying the relationships of air pollution and diabetes are increasing (Honda et al., 2017, Renzi et al., 2018, Strak et al., 2017). Some studies reported positive associations while others detected negative or null associations (Coogan et al., 2016, Eze et al., 2017). For example, To et al. (2015) concluded PM_2.5_ was associated with a 28% increase in prevalence rate ratios (PRs) of diabetes among females (a 10 μg/m^3^ increment, PRs = 1.28, 95%CIs: 1.16, 1.41). However, Strak et al. (2017) did not find a significant result (PM_2.5_: ORs = 1.01, 95%CIs: 0.99, 1.03). Inconsistent findings were also reported in several meta-analyses. For example, Balti et al. (2014) reviewed PM_2.5_ and NO_2_ were positively related to increased type 2 diabetes incidence (PM_2.5_: hazard ratios (HRs) = 1.11, 95%CIs: 1.03, 1.20; NO_2_: HRs = 1.13, 95%CIs: 1.01, 1.22), while another meta-analysis by Eze et al. found the association was only statistically significant among females (Eze et al., 2015). In addition, studies mentioned above mainly focused on the effect of PM_2.5_ and NO_2_ in urban areas of high-income countries in North America and Europe. Thus, it is highly necessary to assess the relationships of PM_1_, PM_2.5_, NO_2_ and type 2 diabetes at high exposure levels in low-/middle income countries.

The purpose of our study is to evaluate the associations between residential exposure to PM_1_, PM_2.5_, NO_2_ and type 2 diabetes prevalence and fasting blood glucose levels in Chinese populations. Also, potential modifying factors in the associations were investigated.

## 2 Material and methods

### 2.1 Populations

**Fig. 1:**
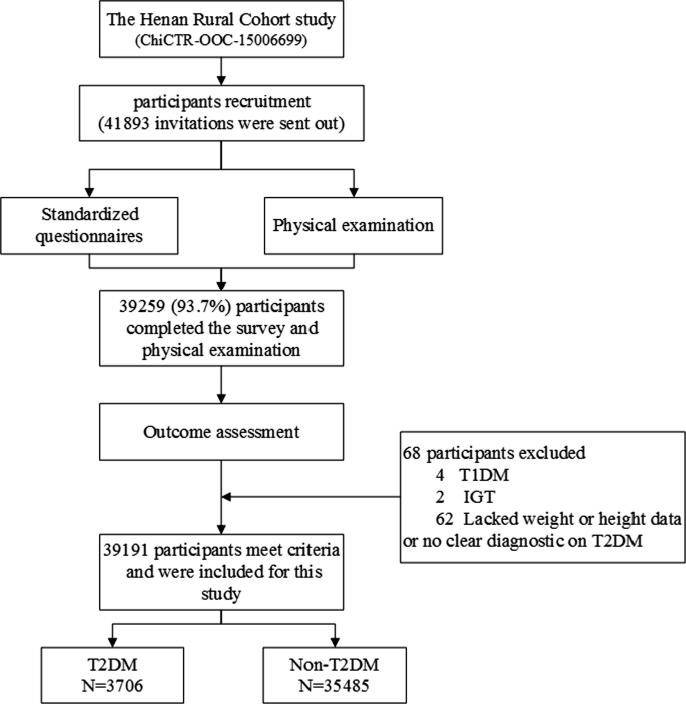
The flowchart of participants recruitment of this present study.

Populations from the rural areas (aged 18–79 years) of Henan province in China were enrolled (Liu et al., 2019b, Tian et al., 2018). We conducted standardized questionnaire surveys by professional public health researchers and medical examinations by physicians between July 2015 and September 2017. Detailed information about the study designs and eligibility criteria has been described previously (Liu et al., 2017, Liu et al., 2019b). A total of 39, 259 people completed questionnaires and accepted medical examinations (Li et al., 2019b). Among these subjects, 4 participants with type 1 diabetes mellitus, 2 with impaired glucose tolerance, and 62 without weight or height data or clear diagnosis on type 2 diabetes were excluded. Ultimately, 39, 191 participants were selected for the current analysis (Fig. 1).

### 2.2 Data collection

Baseline information including age, sex, demographic characteristics, lifestyle and other health-related information were collected from the standardized questionnaire. Demographic characteristics included living region, education level (elementary school or below, middle school, high school or above), marital status (married/living together, divorced/widowed/separated, unmarried), average monthly income (<500, 500–999, ≥1000 RMB). Lifestyle information included: smoking (never, former, current), alcohol drinking (never, former, current), fruit and vegetable intake (an average intake of fruit and vegetable by each participant more than 500 g per day) (no, yes), and physical activity (low, moderate, high). Physical activity was measured in accordance with the international physical activity questionnaire (Craig et al., 2003). Other health-related information included family history of diabetes mellitus (no, yes) and current type 2 diabetes medicines usage (no, yes). Height and weight of each subject were measured twice and the average of these measurements was taken.

### 2.3 Outcome assessments

Blood samples were collected after at least 8-hour overnight fasting. Plasma and serum were immediately separated by centrifugation, and then sent for biochemical analyses. In the meanwhile, fasting blood glucose levels of participants were determined by the automatic biochemical analyzer using the glucose oxidative method. Individuals in our research were diagnosed as type 2 diabetes if they had been previously diagnosed with type 2 diabetes and currently use antidiabetic medicines (such as acarbose, insulin and metformin) and/or had fasting blood glucose levels exceeding 7.0 mmol/L (Tian et al., 2018). Exclusion criteria included type 1 diabetes mellitus, gestational diabetes mellitus, impaired fasting glucose and impaired glucose tolerance (American Diabetes, 2009, Liu et al., 2018).

### 2.4 Exposure estimates

A satellite-based spatiotemporal model was employed to estimate individual exposures to PM_1_, PM_2.5_ and NO_2_ at a 0.1°×0.1° spatial resolution. Briefly, daily data from ground monitoring and aerosol optical depth data with spatial and temporal predictors were combined to estimate the concentrations of PM_1_, PM_2.5_, NO_2_ (Chen et al., 2018b). By comparing predicted data with ground-level measurements of air pollutants, the spatial temporal model showed a good predictive ability (Zhang et al., 2019). The performance of model and accuracy of estimation for daily and annual average PM_1_ were 55% and 20.5 µg/m^3^, and 75% and 8.8 µg/m^3^, respectively (Chen et al., 2018a). Those for PM_2.5_ were 83% and 18.1 µg/m^3^ and 86% and 6.9 μg/m^3^ respectively (Chen et al., 2018c). The performance of model and accuracy of estimation for daily NO_2_ predictions were 62% and 13.3 µg/m^3^ (Zhang et al., 2019). Residential addresses of participants were geocoded using GPSspgxGeocoding software (http://www.gpsspg.com/xgeocoding/download/), which can analyze the longitude and latitude of residential addresses automatically. We matched estimates of air pollutants concentrations to each participant by the geocoded residential addresses. The 3-year average concentrations of PM_1_, PM_2.5_ and NO_2_ for each participant was calculated to estimate the long-term air pollution exposure.

### 2.5 Statistical analysis

The relationship between any two pollutants was assessed using the test of Pearson correlation. We calculated ORs with 95%CIs for each 1 μg/m^3^ increase in single pollutant concentrations to evaluate the relationships of PM_1_, PM_2.5_, NO_2_ and prevalent type 2 diabetes using the logistic regression model. Meanwhile, we used the linear regression model to calculate the regression coefficient with 95%CIs to examine the relationships of PM_1_, PM_2.5_, NO_2_ and fasting blood glucose levels. Two types of model (Model 1 and Model 2) were performed in our analyses. The effect of each pollutant was evaluated separately. Among the two models, model 1 was the preliminary model (adjusted for age and sex) and model 2 was controlled for age, sex, education levels, marital status, the average monthly income, smoking, drinking, high-fat diet, fruit and vegetable intake, physical activity, family history of diabetes and BMI.

Subgroup analyses stratified by age (<65 *v.s.* ≥65 years) and sex (male *v.s.* female) were performed. The statistical difference between the subgroups was tested by including an interaction term. In addition, simple stratified analyses were also performed to verify the results. We finally conducted sensitivity analyses to examine the robustness of our results: (1) we additionally adjusted for region to test the potential effect of spatial clustering in the association. (2) we excluded all type 2 diabetes patients to evaluate the robustness of the estimated associations between PM_1_, PM_2.5_, NO_2_ exposure and fasting blood glucose levels, in order to eliminate the mediating effect of type 2 diabetes on fasting blood glucose levels. All the analyses in our study were performed in SAS.

## 3 Results

Table 1 and Supplementary Table S1 show characteristics of potential risk factors of participants and individual exposure to air pollutants (PM_1_, PM_2.5_, NO_2_) in the study. The studied population had an average age of 55.6 years, 39.4% of them were male. Prevalence of type 2 diabetes in rural residents was 9.5%. Comparing with people without type 2 diabetes, type 2 diabetes patients were significantly older (60.3 year *v.s.* 55.1 years, *p* < 0.01), eat fewer fruit and vegetable (35.7% *v.s.*42.4%, *p* < 0.01), with a family history of diabetes (10.0% *v.s.* 3.6%, *p* < 0.01) and had a higher BMI (26.2 kg/m^2^ *v.s.* 24.7 kg/m^2^, *p* < 0.01). The mean exposure concentration of PM_1_, PM_2.5_ and NO_2_ was 57.4 μg/m^3^, 73.4 μg/m^3^, 39.9 μg/m^3^, respectively. The 3-year average air pollutants exposure levels were higher among type 2 diabetes patients than people without type 2 diabetes (*p* < 0.01). PM_1_ and PM_2.5_ exposure levels were well correlated with NO_2_ (Pearson correlation coefficients = 0.782, 0.899). The correlation coefficients between PM_1_ and PM_2.5_ reached 0.932.

**Table 1:** Characteristics of socio-demographic and major risk factors of participants in the rural areas of China.

Characteristics	Total	Individuals without type 2 diabetes	Type 2 diabetes patients	*P*-value#
N (%)	39,191	35,485 (90.5)	3706 (9.5)	–
**FBG (mmol/L), mean ± SD**	5.5 ± 1.5	5.2 ± 0.6	8.9 ± 2.9	<0.01
PM_1_ (μg/m^3^), mean ± SD	57.4 ± 2.7	57.4 ± 2.7	57.8 ± 2.7	<0.01
PM_2.5_ (μg/m^3^), mean ± SD)	73.4 ± 2.6	73.4 ± 2.6	73.9 ± 2.5	<0.01
NO_2_ (μg/m^3^), mean ± SD	39.9 ± 3.6	39.8 ± 3.6	40.6 ± 3.5	<0.01
Age (year), mean ± SD	55.6 ± 12.2	55.1 ± 12.3	60.3 ± 9.3	<0.01
Age < 65	28,863 (73.6)	26,482 (74.6)	2381 (64.2)	<0.01
Age ≥ 65	10,328 (26.4)	9003 (25.4)	1325 (35.8)	
Sex, n (%)				
Male	15,460 (39.4)	14,049 (39.6)	1411 (38.1)	0.072
Female	23,731 (60.6)	21,436 (60.4)	2295 (61.9)	
Education level, n (%)				
Elementary school or below	17,548 (44.8)	15,499 (43.7)	2049 (55.3)	<0.01
Middle school	15,613 (39.8)	14,390 (40.6)	1223 (33.0)	
High school or above	6030 (15.4)	5596 (15.8)	434 (11.7)	
Marital status, n (%)				
Married/living together	35,185 (89.8)	31,902 (89.9)	3283 (88.6)	<0.01
Divorced/widowed/separated	3399 (8.7)	3000 (8.5)	399 (10.8)	
Unmarried	607 (1.5)	583 (1.6)	24 (0.6)	
Average monthly income, n (%)				
<500 RMB	13,984 (35.7)	12,522 (35.3)	1462 (39.4)	<0.01
500–1000 RMB	12,894 (32.9)	11,703 (33.0)	1191 (32.1)	
>1000 RMB	12,313 (31.4)	11,260 (31.7)	1053 (28.4)	
Smoking, n (%)				
Never	28,533 (72.8)	25,744 (72.5)	2789 (75.3)	<0.01
Former	3185 (8.1)	2808 (8.0)	377 (10.1)	
Current	7473 (19.1)	6933 (19.5)	540 (14.6)	
Drinking, n (%)				
Never	30,295 (77.3)	27,373 (77.1)	2922 (78.8)	<0.01
Former	1828 (4.7)	1589 (4.5)	239 (6.5)	
Current	7068 (18.0)	6523 (18.4)	545 (14.7)	
High-fat diet (≥75 g/day), n (%)				
NO	31,720 (80.9)	28,614 (80.6)	3106 (83.8)	<0.01
YES	7471 (19.1)	6871 (19.4)	600 (16.2)	
Fruit and vegetable intake (≥500 g/day), n (%)*				
NO	22,826 (58.2)	20,445 (57.6)	2381 (64.3)	<0.01
YES	16,363 (41.8)	15,039 (42.4)	1324 (35.7)	
Physical activity, n (%)				
Low	12,682 (32.4)	11,220 (31.6)	1462 (39.4)	<0.01
Moderate	14,788 (37.7)	13,481 (38.0)	1307 (35.3)	
High	11,721 (29.9)	10,784 (30.4)	937 (25.3)	
Family history of diabetes, n (%)				
NO	37,551 (95.8)	34,215 (96.4)	3336 (90.0)	<0.01
YES	1640 (4.2)	1270 (3.6)	370 (10.0)	
BMI (kg/m^2^), mean ± SD	24.8 ± 3.6	24.7 ± 3.5	26.2 ± 3.7	<0.01

* Missing partial data.

# Chi-square tests for categorical variables and t-tests for continuous variables.

**Fig. 2:**
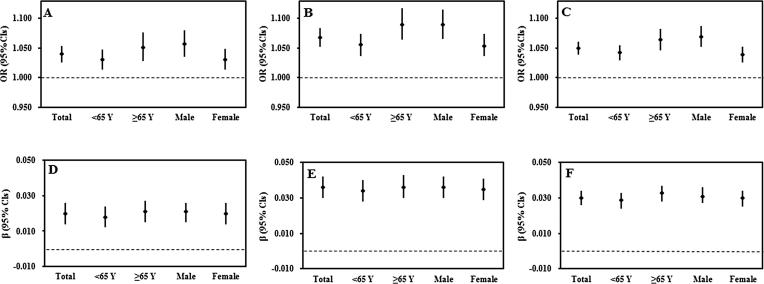
Associations between each 1 μg/m^3^ increase in 3-year average air pollution (PM_1_, PM_2.5_, NO_2_) exposure and type 2 diabetes prevalence and fasting blood glucose levels: Association of PM_1_, PM_2.5_ and NO_2_ exposure with type 2 diabetes prevalence were separately shown in figure A, B and C; Association of PM_1_, PM_2.5_ and NO_2_ exposure with fasting blood glucose levels were separately shown in figure D, E and F.

Table 2 summarizes the relationships of PM_1_, PM_2.5_, NO_2_ exposure and type 2 diabetes prevalence and fasting blood glucose levels. Higher PM_1_, PM_2.5_, NO_2_ concentrations were strongly related to higher odds of type 2 diabetes and higher fasting blood glucose levels in the model 1. Results were significant in the model 2 as well. We found every 1 μg/m^3^ increase in PM_1_, PM_2.5_ and NO_2_ exposure concentrations was related to a 4.0% (95%CIs: 1.026, 1.054), 6.8% (95%CIs: 1.052, 1.084), 5.0% (95%CIs: 1.039, 1.061) increase in odds of type 2 diabetes, and a 0.020 mmol/L (95%CIs:0.014, 0.026), 0.036 mmol/L (95%CIs: 0.030, 0.042) and 0.030 mmol/L (95%CIs:0.026, 0.034) higher fasting blood glucose levels, respectively. (Fig. 2).

**Table 2:** Associations of long-term air pollution exposures with type 2 diabetes prevalence and fasting blood glucose levels per 1 μg/m^3^ increase in exposure.

Air pollutants	Type 2 diabetes prevalence	Fasting blood glucose levels (mmol/L) (mmol/L)
	OR (95%CIs)	β (95% CIs)
**PM_1_ (μg/m^3^)**		
Model 1	1.064 (1.051, 1.078)	0.034 (0.029, 0.040)
Model 2	1.040 (1.026, 1.054)	0.020 (0.014, 0.026)

**PM_2.5_ (μg/m^3^)**		
Model 1	1.096 (1.081, 1.110)	0.052 (0.046, 0.058)
Model 2^a^	1.068 (1.052, 1.084)	0.036 (0.030, 0.042)

**NO_2_ (μg/m^3^)**		
Model 1	1.070 (1.060, 1.080)	0.042 (0.037, 0.046)
Model 2	1.050 (1.039, 1.061)	0.030 (0.026, 0.034)

Model 1: adjusted for age, sex.

Model 2: adjusted for age, sex, education level, marital status, average monthly income, smoking, drinking, high fat diet, fruit and vegetable intake, physical activity, family history of diabetes, BMI.

Table 3 and Supplementary Table S2 displayed the associations between PM_1_, PM_2.5_, NO_2_ and type 2 diabetes prevalence and fasting blood glucose levels stratified by age and sex. There was no significant interaction effect of associations between PM_1_ exposure and type 2 diabetes prevalence by age and sex. However, stronger associations between PM_2.5_, NO_2_ and type 2 diabetes prevalence were presented in individuals ≥65 years and males. Age showed significant interaction effect on the relationships of PM_1_, PM_2.5_, NO_2_ and fasting blood glucose levels (*p* < 0.001). Greater changes of fasting blood glucose levels were found in residents ≥65 years. Interaction effect of NO_2_ exposure and sex on fasting blood glucose levels was significant, while no significant interaction effect was observed for sex on the relationships of PM_1_, PM_2.5_ and fasting blood glucose levels (Fig. 2).

**Table 3:** Interaction effects of covariates in associations between long-term air pollution exposures and type 2 diabetes prevalence and fasting blood glucose levels.

Group	Type 2 diabetes prevalence		Fasting blood glucose levels (mmol/L)	
	Interation OR (95%CIs)	P-value for the interaction	Interation β (95%CIs)	P-value for the interaction
**PM_1_**				
Age^a^				
<65	1.030 (1.014, 1.047)		0.018 (0.012, 0.024)	
≥65	1.051 (1.028, 1.076)	0.150	0.021 (0.015, 0.027)	<0.001
Sex^b^				
Male	1.057 (1.035, 1.080)		0.021 (0.015, 0.026)	
Female	1.030 (1.013, 1.048)	0.058	0.020 (0.014, 0.026)	0.108

**PM_2.5_**				
Age^a^				
<65	1.056 (1.037, 1.074)		0.034 (0.028, 0.040)	
≥65	1.090 (1.064, 1.117)	0.029	0.036 (0.030, 0.043)	<0.001
Sex^b^				
Male	1.090 (1.066, 1.115)		0.036 (0.030, 0.042)	
Female	1.054 (1.036, 1.074)	0.022	0.035 (0.029, 0.041)	0.065

**NO_2_**				
Age^a^				
<65	1.042 (1.029, 1.055)		0.029 (0.024, 0.033)	
≥65	1.064 (1.046, 1.082)	0.046	0.033 (0.028, 0.037)	<0.001
Sex^b^				
Male	1.069 (1.052, 1.087)		0.031 (0.027, 0.036)	
Female	1.039 (1.026, 1.052)	0.005	0.030 (0.025, 0.034)	0.020

^a^ Adjusted for sex, education level, marital status, average monthly income, smoking, drinking, high fat diet, fruit and vegetable intake, physical activity, family history of diabetes, BMI.

b Adjusted for age, education level, marital status, average monthly income, smoking, drinking, high fat diet, fruit and vegetable intake, physical activity, family history of diabetes, BMI.

Supplementary Table S3 and Supplementary Table S4 demonstrated results of sensitivity analyses. The relationships of PM_1_, PM_2.5_, NO_2_ and type 2 diabetes prevalence and fasting blood glucose levels remained significant after additionally adjusted for region. Besides, exclusion of type 2 diabetes patients did not significantly change the effect of PM_1_, PM_2.5_, NO_2_ on fasting blood glucose levels. It showed a 1 μg/m^3^ increase in PM_1_ was related to a 0.011 mmol/L (95%CIs: 0.009, 0.014) higher fasting blood glucose levels, and for PM_2.5_ and NO_2_, the increment of fasting blood glucose levels was 0.020 mmol/L (95%CIs: 0.017, 0.022) and 0.019 mmol/L (95%CIs: 0.017, 0.020), respectively.

## 4 Discussion

Our study is one of few studies addressing the relationships between PM_1_, PM_2.5_, NO_2_ and type 2 diabetes in Chinese rural populations. In general, higher PM_1_, PM_2.5_, NO_2_ exposure concentrations were associated with increased odds of type 2 diabetes and fasting blood glucose levels. And we found the relationships of PM_2.5_, NO_2_ and type 2 diabetes prevalence were stronger in individuals aged 65 years or older and males. The relationships between PM_1_, PM_2.5_, NO_2_ and fasting blood glucose levels remained significant after excluding type 2 diabetes patients.

PM_1_ was positively related to type 2 diabetes prevalence in our present study. Although very few studies focus on PM_1_ exposure, findings of our study were consistent with the existing evidence. A study by Yang et al. concluded an increase in PM_1_ (Per IQR, 15 µg/m^3^) was related to higher odds for the prevalence of type 2 diabetes (ORs = 1.13, 95%CIs: 1.04, 1.22) (Yang et al., 2018a). Moreover, higher exposure level of PM_1_ was also reported to be associated with increased risk of cardiometabolic disease, which may contribute to the incidence of type 2 diabetes. For example, a 10 µg/m^3^ increase in PM_1_ was related to 12% higher odds of metabolic syndrome (ORs = 1.12, 95%CIs: 1.00, 1.24) (Yang et al., 2018b) and 36% higher odds of hyperbetalipoproteinemia (ORs = 1.36, 95%CIs: 1.03, 1.78) in individuals living in Northeastern China (Yang et al., 2019). All above studies suggested adverse effects of PM_1_ on human disease.

Positive relationships of PM_2.5_, NO_2_ and type 2 diabetes prevalence identified in our present study were consistent with previous evidence (Honda et al., 2017, Orioli et al., 2018, Shin et al., 2019, Yang et al., 2018a). However, some other studies reported null associations (Lazarevic et al., 2015, Renzi et al., 2018, Strak et al., 2017). A study conducted in Australia reported NO_2_ was not associated with diabetes (self-reported diabetes) (RRs = 1.04, 95%CIs: 0.90, 1.20) (Lazarevic et al., 2015). Besides, a study performed in the Netherlands reviewed that the relationship of PM_2.5_ and prevalent diabetes (self-reported diabetes and/or use of diabetes medication) was non-significant (ORs = 1.01, 95%CIs: 0.99, 1.03) (Strak et al., 2017). Inconsistencies may be due to factors including differences in inclusion criteria, study regions, population characteristics, exposure patterns, exposure measurements, chemical compositions of air pollutants.

The associations of PM_1_ with type 2 diabetes prevalence and fasting blood glucose variations were weaker than the association found for PM_2.5_. For instance, every 1 μg/m^3^ increase in PM_2.5_ and PM_1_ was respectively related to 6.8% and 4.0% higher odds of type 2 diabetes. Possible reasons for this difference include distinct sources of PM_2.5_ and PM_1_ and different proportion of chemical components. For example, at the Yinglite (38°19′N, 106°67′E), elemental carbon (EC), organic matter (OM), water-soluble inorganic ions, and mineral dust contributed 5.0%, 10.5%, 20.4%, and 28.3% to PM_2.5_ mass, while these chemical components accounted for 6.5%, 15.0%, 29.2%, and 45.2% of PM_1_ mass (Liang et al., 2019). Moreover, those chemical components of PM_2.5_ and PM_1_ may also have an important function in type 2 diabetes development, which affect the effect magnitude of PM_2.5_ and PM_1_ on type 2 diabetes.

Positive relationships of PM_1_, PM_2.5_, NO_2_ and fasting blood glucose levels were identified in our study. Similar findings were also illustrated in other previous studies in China (Chen et al., 2016, Tan et al., 2018, Yang et al., 2018a). However, some studies in Europe reported inconsistent findings. For example, Riant et al. (2018) found relationships of NO_2_ and fasting blood glucose levels were statistically insignificant in Northern France (NO_2_: β = 0.0046, 95%CIs: −0.0024, 0.0115). We noted that the level of NO_2_ in Riant’s study was lower than the WHO’s guideline value (21.96 *v.s.* 40 μg/m^3^). However, the concentrations of air pollutants were much higher than the WHO guideline value in China. This low exposure level of air pollutants in France may explain the non-significant association. There may also be other reasons for the inconsistency. Studies are needed to examine threshold for the effect of air pollutants on fasting blood glucose levels.

Significant relationships of PM_2.5_, NO_2_ and prevalent type 2 diabetes were apparent in the residents aged 65 years or older and in males. For PM_1_, the interaction with age and sex were not significant. Our results were generally consistent with other studies, but some differences remained. For example, a study in Hong Kong reported that the associations of long-term exposure to PM_2.5_ with prevalence of type 2 diabetes were only statistically significant among females (Qiu et al., 2018), while another study in northeastern China showed significant associations of long-term exposure to PM_1_, PM_2.5_ with type 2 diabetes were mainly apparent for the young adults (<50 years of age) and females (Yang et al., 2018a). However, a recent meta-analysis indicated that no significant difference in associations of PM_2.5_, NO_2_ with type 2 diabetes prevalence between males and females (Liu et al., 2019a). Thus, difference in sensitivity between females and males towards air pollution remains unknown.

Potential mechanisms of type 2 diabetes development and air pollution are still unclear. One potential mechanism is that air pollutants may result in systemic inflammation, which lead to abnormalities of insulin signaling and disbalance of glucose homeostasis (Brook et al., 2010). Sun et al. (2009) first reported that air pollution exposure can increase insulin resistance and visceral inflammation in a mouse model. Liu et al. (2014) then found that air pollution exposure increased inflammation in insulin target tissues and resulted in an impaired energy metabolism in a genetically susceptible diabetic model. Recently, alterations in inflammation marker after air pollution exposure have been reported by numerous studies, though most studied pollutants only include PM_2.5_, PM_10_ and NO_2_ (Li et al., 2019a, Li et al., 2019c, Lucht et al., 2019). Studies on the mechanism of PM_1_ is less. Valavanidis et al. (2008) indicated that the smaller size of particulate matter the higher toxicity related to mechanisms of inflammation. A study of Zhou et al reported the contribution of organic aerosols (OA) and secondary inorganic aerosols to PM_1_ was 53.0% and 35.0% to the mass, respectively (Zhou et al., 2019). The potential of inflammation may be mainly caused by those aerosol species of PM_1_. Further studies are needed to clarify the underlying mechanisms for the associations and to explore how to reduce the risk of type 2 diabetes.

Limitations existed in our study. Firstly, only fasting blood glucose was used for glucose measurements. The glycated hemoglobin A1c (HbA1c) is a more accurate indicator to identify individual’s long-term mean blood glucose levels, but it was not measured in the present study. Lack of measurement of HbA1c may lead the underestimation of effect size (Riant et al., 2018). Secondly, fasting blood glucose in our study was measured only once for each participant according to the study design, which may cause random measurement error to some extent. Thirdly, we were unable to control other potential confounders such as green space, traffic and noise exposures and indoor air pollution exposures, due to unavailability of these information in the survey (Clark et al., 2017, Faizan and Thakur, 2019). The omission of those potential confounders may affect the effect magnitude of air pollution on diabetes. Moreover, findings of this study cannot indicate the health effect of exposure to a specific air pollutant because of exposure mixture and high correlation of muti-pollutants, although the present discussion mainly focused on PM_1_.

## 5 Conclusion

Our findings suggest that higher exposure concentrations of PM_1_, PM_2.5_, NO_2_ were related to higher odds of type 2 diabetes and fasting blood glucose levels in the Chinese rural population. Future prospective studies with broader geographic areas are still needed to verify our results and to confirm the relationship between PM_1_ and PM_2.5_.
