# Supplementary material for: Associations of long-term exposure to PM1, PM2.5, NO2 with type 2 diabetes mellitus prevalence and fasting blood glucose levels in Chinese rural populations
Source: Environ Int. 2019 Dec;133(Pt B):105213. doi: 10.1016/j.envint.2019.105213 (PMC6853163; doi:10.1016/j.envint.2019.105213)
Supplement: Supplementary data 2 [file mmc2.docx]

**Supplementary Material**

Supplementary Table S1. Characteristics of air pollutants of participants

| pollutants | Mean | SD | Min | P_25_ | P_50_ | P_75_ | Max | IQR |
| --- | --- | --- | --- | --- | --- | --- | --- | --- |
| PM_1_ (μg/m^3^) | 57.4 | 2.7 | 48.1 | 55.5 | 57.0 | 59.0 | 70.9 | 3.9 |
| PM_2.5_ (μg/m^3^) | 73.4 | 2.6 | 68.0 | 71.5 | 73.3 | 76.0 | 84.9 | 3.0 |
| NO_2_ (μg/m^3^) | 39.9 | 3.6 | 31.0 | 36.3 | 40.3 | 42.7 | 49.8 | 6.6 |

Supplementary Table S2. Simple stratified analysis of the associations between long-term air pollution exposures and type 2 diabetes prevalence and fasting blood glucose levels

| Subgroup | Type 2 diabetes prevalence |  | Fasting blood glucose levels (mmol/L) |
| --- | --- | --- | --- |
|  | OR (95%CIs) |  | β (95%CIs) |
| **PM_1_** |  |  |  |
| Age^b^ |  |  |  |
| <65 | 1.036 (1.019, 1.053) |  | 0.016 (0.010, 0.032) |
| >=65 | 1.052 (1.027, 1.077) |  | 0.029 (0.017, 0.042) |
| Sex^c^ |  |  |  |
| Male | 1.050 (1.027, 1.074) |  | 0.031 (0.022, 0.041) |
| Female | 1.036 (1.018, 1.054) |  | 0.014 (0.007, 0.021) |
| **PM_2.5_** |  |  |  |
| Age^b^ |  |  |  |
| <65 | 1.062 (1.044, 1.081) |  | 0.031 (0.024, 0.038) |
| >=65 | 1.091 (1.063, 1.120) |  | 0.051 (0.038, 0.063) |
| Sex^c^ |  |  |  |
| Male | 1.083 (1.058, 1.109) |  | 0.048 (0.039, 0.058) |
| Female | 1.060 (1.041, 1.080) |  | 0.028 (0.021, 0.036) |
| **NO_2_** |  |  |  |
| Age^b^ |  |  |  |
| <65 | 1.047 (1.034, 1.061) |  | 0.028 (0.023, 0.033) |
| >=65 | 1.064 (1.045, 1.083) |  | 0.038 (0.029, 0.047) |
| Sex^c^ |  |  |  |
| Male | 1.064 (1.046, 1.082) |  | 0.039 (0.032, 0.046) |
| Female | 1.044 (1.031, 1.058) |  | 0.025 (0.020, 0.031) |

^b^ adjusted for sex, education level, marital status, average monthly income, smoking, drinking, high fat diet, fruit and vegetable intake, physical activity, family history of diabetes, BMI.

^c^ adjusted for age, education level, marital status, average monthly income, smoking, drinking, high fat diet, fruit and vegetable intake, physical activity, family history of diabetes, BMI.

Supplementary Table S3. Sensitivity analyses of the associations of long-term air pollution exposures with type 2 diabetes prevalence and fasting blood glucose levels per 1 ug/m^3^ increase in exposure

| Air pollutants | Type 2 diabetes prevalence |  | Fasting blood glucose levels (mmol/L) (mmol/L) |
| --- | --- | --- | --- |
|  | OR^*^ (95%CIs) |  | β^*^ (95% CIs) |
| PM_1_ | 1.049 (1.035, 1.064) |  | 0.025 (0.019, 0.031) |
| PM_2.5_ | 1.068 (1.052, 1.084) |  | 0.031 (0.025, 0.037) |
| NO_2_ | 1.051 (1.040, 1.062) |  | 0.028 (0.023, 0.032) |

^*^ Adjusted for age, sex, education level, marital status, average monthly income, smoking, drinking, high fat diet, fruit and vegetable intake, physical activity, family history of diabetes, BMI, **region**.

Supplementary Table S4. Sensitivity analyses of the association between air pollutants exposure and fasting blood glucose levels in individuals without type 2 diabetes

| Group | Absolut change of of fasting blood glucose in mmol/L | |
| --- | --- | --- |
|  | β (95%CIs) | P-value for the interaction |
| **PM_1_** |  |  |
| Adjusted model^a^ | 0.011 (0.009, 0.014) | - |
| Age^b^ |  |  |
| <65 | 0.010 (0.008, 0.013) |  |
| >=65 | 0.012 (0.009, 0.015) | < 0.001 |
| Sex^c^ |  |  |
| Male | 0.012 (0.009, 0.014) |  |
| Female | 0.011 (0.009, 0.014) | 0.377 |
| **PM_2.5_** |  |  |
| Adjusted model^a^ | 0.020 (0.017, 0.022) | - |
| Age^b^ |  |  |
| <65 | 0.019 (0.016, 0.021) |  |
| >=65 | 0.020 (0.018, 0.023) | < 0.001 |
| Sex^c^ |  |  |
| Male | 0.020 (0.018, 0.022) |  |
| Female | 0.020 (0.017, 0.022) | 0.211 |
| **NO_2_** |  |  |
| Adjusted model^a^ | 0.019 (0.017, 0.020) | - |
| Age^b^ |  |  |
| <65 | 0.018 (0.016, 0.019) |  |
| >=65 | 0.020 (0.018, 0.022) | < 0.001 |
| Sex^c^ |  |  |
| Male | 0.019 (0.017, 0.020) |  |
| Female | 0.018 (0.017, 0.020) | 0.033 |

^a^ adjusted for age, sex, education level, marital status, average monthly income, smoking, drinking, high fat diet, fruit and vegetable intake, physical activity, family history of diabetes, BMI.

^b^ adjusted for sex, education level, marital status, average monthly income, smoking, drinking, high fat diet, fruit and vegetable intake, physical activity, family history of diabetes, BMI.

^c^ adjusted for age, education level, marital status, average monthly income, smoking, drinking, high fat diet, fruit and vegetable intake, physical activity, family history of diabetes, BMI.
